# Maternal diabetes in pregnancy and offspring cognitive ability: sibling study with 723,775 men from 579,857 families

**DOI:** 10.1007/s00125-013-3065-z

**Published:** 2013-09-25

**Authors:** Abigail Fraser, Catarina Almqvist, Henrik Larsson, Niklas Långström, Debbie A. Lawlor

**Affiliations:** 1 MRC Integrative Epidemiology Unit at the University of Bristol, Oakfield House, Oakfield Grove, Bristol, BS8 2BN UK; 2 Department of Medical Epidemiology and Biostatistics, Karolinska Institutet, Stockholm, Sweden; 3 Lung and Allergy Unit, Astrid Lindgren Children’s Hospital, Karolinska University Hospital, Stockholm, Sweden

**Keywords:** Cognition, Diabetes, IQ, Pregnancy, Sibling comparison, Swedish registers

## Abstract

**Aims/hypothesis:**

The aim of this study was to investigate the association between maternal diabetes in pregnancy and offspring cognitive ability and also to assess whether the association was due to intrauterine mechanisms or shared familial characteristics.

**Methods:**

We linked national registers and conducted a prospective cohort study of singleton Swedish-born men to explore associations between maternal pregnancy diabetes and educational achievement at age 16 years, the age of completing compulsory education in Sweden (*n* = 391,545 men from 337,174 families, graduating in 1988–1997 and *n* = 326,033 men from 282,079 families, graduating in 1998–2009), and intelligence quotient (IQ) at the mandatory conscription examination at 18 years of age (*n* = 664,871 from 543,203 families).

**Results:**

Among non-siblings, maternal diabetes in pregnancy was associated with lower offspring cognitive ability even after adjustment for maternal age at birth, parity, education, early-pregnancy BMI, offspring birth year, gestational age and birthweight. For example, in non-siblings, the IQ of men whose mothers had diabetes in their pregnancy was on average 1.36 points lower (95% CI −2.12, −0.60) than men whose mothers did not have diabetes. In comparison, we found no such association within sibships (mean difference 1.70; 95% CI −1.80, 5.21).

**Conclusions/interpretation:**

The association between maternal diabetes in pregnancy and offspring cognitive outcomes is likely explained by shared familial characteristics and not by an intrauterine mechanism.

## Introduction

A number of studies comparing cognitive measures in offspring of mothers who had diabetes in pregnancy with offspring of mothers who did not, report lower scores on some indices but not lower overall intelligence quotient (IQ) in offspring exposed to maternal diabetes in western populations [1–6]. The largest epidemiological study to date used population registry data from Sweden (*n*∼1.3 million adolescents) and found that maternal pregnancy diabetes was associated with lower offspring educational achievement at age 16 years [7]. A recent report based on data from the UK-based Avon Longitudinal Study of Parents and Children (ALSPAC) found that exposure to pre-existing maternal diabetes and gestational diabetes were both associated with lower IQ measured in childhood (age 8 years) and that existing diabetes was also associated with lower educational achievement at 16 years [8]. Whereas the results of the earlier Swedish registry-based study may be explained by residual confounding due to the unavailability of data on potential confounders [7], the recent ALSPAC analyses could adjust for a wide range of confounders but did so at the cost of limited statistical power [8]. In contrast to these two studies from Western populations, where diabetes in pregnancy is relatively uncommon (0.5% [7]), a recent study from India which reported a 7% prevalence of gestational diabetes [9], suggested that gestational diabetes was associated with higher cognitive achievement scores in offspring at a mean age of 9.7 years [10].

**Fig. 1:**
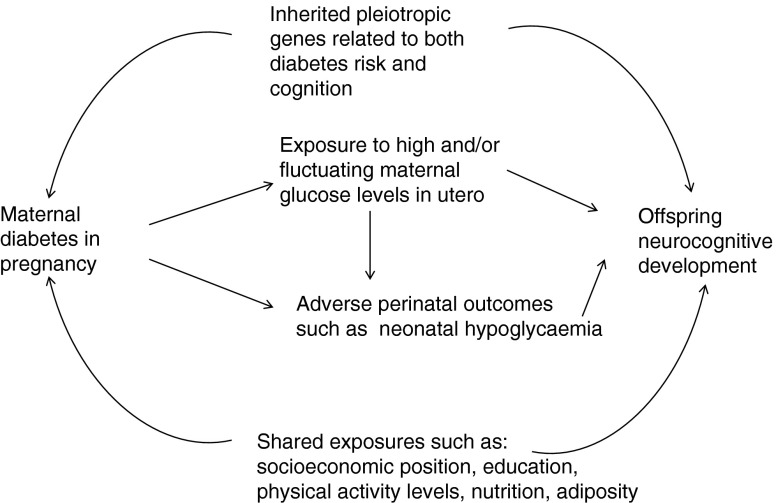
Schematic representation of potential pathways linking maternal diabetes in pregnancy and offspring cognition

Several mechanisms could explain the association between exposure to diabetes in utero and poorer cognitive outcomes (see Fig. 1). Maternal diabetes is associated with neonatal complications which may in turn adversely affect neurocognitive and psychomotor development [11, 12]. Maternal diabetes in pregnancy could also affect fetal neurodevelopment via in utero exposure to a metabolic milieu with high or fluctuating concentrations of glucose and, potentially, ketonaemia [13]. In women with pregnancy diabetes, better glucose control has been associated with higher offspring cognitive function [5, 12]. If these mechanisms were true, this would be additional evidence supporting the need to identify and control glucose intolerance in pregnancy and for interventions to reduce BMI in women of reproductive age, as pre-pregnancy BMI is positively associated with the risk of gestational diabetes [14].

Alternatively, pleiotropic effects of genes shared by mother and offspring and related to both risk of diabetes and IQ could result in an association between pregnancy diabetes and offspring cognitive ability. Finally, shared familial environmental exposures, such as socioeconomic position, educational attainment, level of physical activity and nutrition, may be driving the association.

Sibling studies are a form of natural experiment and a powerful design in terms of dealing with unmeasured or poorly measured confounders that are identical or similar in siblings, such as early-life familial characteristics and genetics [15]. If siblings exposed in utero to pregnancy diabetes had poorer cognitive outcomes than their diabetes-unexposed siblings, this would support a causal relationship (either a direct effect due to intrauterine exposure or via perinatal complications, see Fig. 1), since such an association could not be explained by familial socioeconomic position or maternal genotype, which are the same for siblings. In this study, we conducted a sibling study of the association between maternal pregnancy diabetes and offspring cognitive outcomes to identify potential pathways.

## Methods

### Study subjects

We used data from the Swedish national conscription examination for offspring IQ. During the years covered by this study it was legally required that all Swedish young men attended the military service conscription examination; only those with a severe handicap or chronic disease (<5% of the male population) were exempt from the examination [16]. Hence, we included all men born in Sweden between 1973 and 1992, who were still alive and completed their compulsory school education at 16 years of age (between 1988 and 2009) or their conscription medical examination at age 18 years (between 1990 and 2010).

Data on the index participant’s birth date, together with maternal age at birth and the sex and age of full siblings, were extracted from the Multi-Generation Register. We linked these data with the Medical Birth [17, 18], the Inpatient, Military Service Conscription and the Grade 9 School Marks Registers, and the Population and Housing Census of 1990. The Regional Ethics Committee at Karolinska Institutet, Stockholm approved these linkages.

**Fig. 2:**
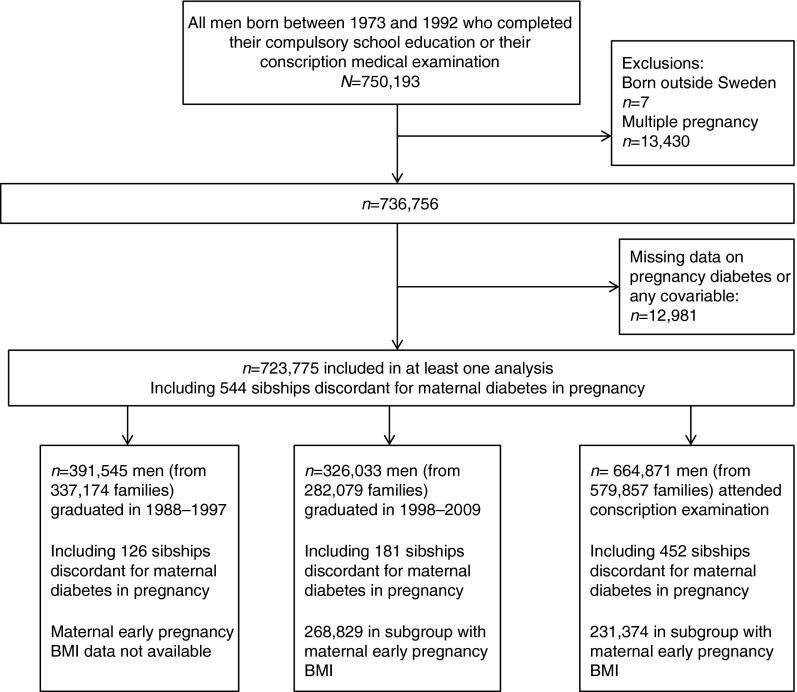
Study flow chart

We excluded anyone born outside of Sweden, multiple births and anyone with missing data on any of the variables used in this study. Figure 2 shows the derivation of the eligible and final analyses cohorts.

### Outcomes

Two outcomes were examined: (1) educational achievement at age 16 years, which is the average age of completing the compulsory 9 year education in Sweden and (2) IQ at the conscription examination at 18 years of age. From 1988 to 1997 school marks in Sweden were numerical and normally distributed on a five-level scale for each subject. The Grade 9 School Mark Registers provides the average mark of all subjects for each student who completed compulsory education in 1988–1997. From 1998, the marking system changed and marks for each student were given on a four-level categorical scale: fail, pass, pass with distinction and pass with excellence. These were assigned values of 0, 10, 15 and 20 in the Register. For each student, the highest 16 marks were then summed to provide a scale ranging from 0 to 320 (where 320 represents the highest mark, 20, × 16) in the Register. Because of this change in the marking system, analyses were conducted separately for the two periods, 1988–1997 and 1998–2009.

IQ was measured at military conscription at a mean age of 17.8 years (SD 0.54 years). The IQ test consisted of four subtests which measured logical, spatial, verbal and technical ability. During the study period two different IQ tests were used to obtain a global IQ score, as described previously in more detail [19]. Briefly, the first test was in use in 1969–1994 and the second test from 1994 onwards. The two tests were similar and the global IQ score for both tests was standardised annually against the entire tested population to follow a Gaussian distribution with values between 1 and 9, with a mean of 5 and an SD of 2, also known as a Stanine scale. These categories, from 1 to 9, are equivalent to IQ bands of less than 74, 74–81, 82–89, 90–95, 96–104, 105–110, 111–118, 119–126 and more than 126, respectively [20]. To analyse IQ in interpretable units, we recorded the nine Stanine score values to the midpoint of the IQ bands.

### Exposure and other variables

Data on maternal diabetes in pregnancy, parity, height and weight at first antenatal clinic assessment, and offspring birthweight were measured or ascertained by midwives, obstetricians or physicians as part of normal clinical practice. Information on these variables was taken directly from the obstetric records and entered into the Medical Birth Register. The Inpatient Register was also used to identify women with diabetes in pregnancy using the following International Classification of Diseases codes: ICD-8, 250 and 761.1 (until 1986); ICD-9, 250, 648.0 and 648.8 (from 1987) (www.icd9data.com/2007/Volume1/240-279/250-259/250/default.htm). Since data in the Medical Birth Register does not distinguish between gestational or existing diabetes, we used the term ‘maternal diabetes in pregnancy’ for this exposure. Maternal early pregnancy weight and height was recorded from 1982, so for the cohort that completed compulsory school education in 1988–1997 these data were unavailable (and, hence, it was not possible to calculate BMI). Maternal early pregnancy was coded as underweight (BMI <18.5 kg/m^2^), normal (≥18.5 and <25 kg/m^2^), overweight (≥25 and <30 kg/m^2^), obese class I (≥30 and <35 kg/m^2^) and obese classes 2 and 3 (≥35 kg/m^2^). The latter classes were collapsed due to limited numbers. Gestational age was assessed from the first day of the last menstrual period for 83% of the cohort, with ultrasound scan results being used alone or in combination with last menstrual period in the remainder. Maternal education (incomplete compulsory school; 9 years compulsory school; upper secondary school; post secondary; postgraduate) was obtained from the 1990 census.

### Statistical analyses

Each model involved fixed effect, between sibling and random effect regression models, generated using the xtreg command in Stata 10 (Stata, Houston, Texas). The fixed-effect regression analysis provides the within-sibship association. This coefficient represents the association of maternal pregnancy diabetes with offspring cognitive ability having controlled for fixed maternal characteristics (e.g. socioeconomic background, lifestyle and genes). An inverse association here supports an intrauterine effect by suggesting that the sibling exposed to diabetes in utero (with all fixed maternal characteristics controlled for) has worse educational achievement and/or lower IQ than the sibling who was not exposed to maternal diabetes in utero. The second regression model obtains the between-non-siblings effect. This coefficient represents the association of maternal pregnancy diabetes with offspring outcomes among non-siblings. The random effects regression coefficients are then obtained as the weighted average of the within-sibling and between-non-sibling effects, each coefficient weighted by the inverse of its variance [21, 22]. This coefficient represents the overall association between the maternal pregnancy diabetes and offspring educational achievement and/or IQ (i.e. without control for fixed maternal characteristics), taking family clustering into account in the estimation of 95% CIs, and is presented for completion. The effect estimates from the within-sibship and between-non-sibling models were compared using a Hausman test in which the null hypothesis assumes that there is no difference between the two estimates.

In the basic model (model 1), we adjusted only for birth year (of all siblings). We then adjusted also for potential confounding by maternal age at birth, parity and education (model 2). It should be noted that maternal education does not contribute in the within-sibling analyses because it is the same for both siblings. We repeated this model, restricting to the subsample for which early-pregnancy BMI was available. This was done to assess whether results for this subsample were representative of the whole study population. In model 3, we also adjusted for maternal early-pregnancy BMI, a key risk factor for gestational diabetes also associated with offspring cognitive ability [23] and, therefore, a possible confounder of the examined association. In model 4, we explored possible mediation of associations by gestational age and birthweight. Maternal diabetes is associated with greater fetal and infant adiposity (at birth) due to intrauterine mechanisms related to fetal insulin secretion [24]. There is also evidence that birthweight is inversely correlated with cognitive outcome [25]. Therefore, we wanted to examine whether any association of maternal pregnancy diabetes with offspring cognitive outcome was explained by greater adiposity at birth that persisted into adulthood. Before adjusting for maternal early-pregnancy BMI, we ascertained that there was no evidence of interaction between maternal early pregnancy and diabetes in pregnancy in their associations with offspring BMI by including interaction terms in the regression models (*p* > 0.64 for all).

## Results

Figure 2 shows the derivation of eligible and final analyses cohorts. Data on maternal diabetes in pregnancy and either educational achievement or IQ at conscription were available for 723,775 men from 579,857 families. In male sibships discordant for exposure to maternal pregnancy diabetes (*n* = 544), 46% (*n* = 249) of first-, 50% (*n* = 272) of second-, 48% (*n* = 50) of third- and 20% (*n* = 2) of fourth- and fifth-order siblings were exposed to maternal pregnancy diabetes in utero. In families with two sons, pregnancy diabetes rates were similar in the first and second pregnancies (49 and 51%, respectively). The correlation between IQ assessed at conscription at age 18 years and educational achievement at age 16 was 0.64 for those who completed compulsory education in 1988–1997 and 0.55 for those who graduated in 1998–2009 (*p* < 0.001 for both). Table 1 compares characteristics of the 723,775 eligible men included in at least one of the analyses with those excluded because of some missing data. There were differences between included and excluded men; these differences were generally small but many were statistically significant at conventional levels of *p* < 0.05 due to the large sample size. Similarly, small differences were noted when we compared the characteristics of men with and without data on maternal early-pregnancy BMI (data not shown but available on request).

**Table 1:** Characteristics of men born in Sweden and included in at least one analysis (*n* = 723,775) and those excluded from all analyses due to missing data

Characteristic	Excluded (*n* with missing data)	Excluded	Included	*p* value
Maternal characteristic				
Diabetes in pregnancy	12,981			
Yes		98 (0.8)	3,526 (0.5)	<0.001
No		12,883 (99.2)	720,249 (99.5)	
Early-pregnancy BMI categories*	4,725			<0.001
Underweight		516 (10.9)	22,341 (8.25)	
Normal		3,506 (74.2)	210,926 (77.9)	
Overweight		622 (13.2)	33,579 (12.4)	
Obese		115 (2.4)	5,465 (2.0)	
Mean early-pregnancy BMI, kg/m^2a^	4,725	21.9 (3.3)	22.0 (3.0)	0.09
Age at birth, years	12,981	27.3 (5.6)	27.3 (5.0)	0.60
Parity	12,981			<0.001
1		5,534 (42.6)	305,167 (42.2)	
2		4,333 (33.4)	270,500 (37.4)	
3		1,957 (15.1)	109,511 (15.1)	
4		683 (5.3)	28,044 (3.9)	
5		261 (2.0)	7,038 (1.0)	
≥6		213 (1.6)	3,515 (0.5)	
Highest education	5,159			<0.001
Not completed compulsory school		466 (9.0)	47,467 (6.6)	
9 year compulsory school		987 (19.1)	115,414 (16.0)	
Upper secondary school		2,493 (48.3)	360,060 (49.7)	
Post secondary		1,205 (23.4)	199,252 (27.5)	
Postgraduate		8 (0.2)	1,582 (0.2)	
Offspring characteristic				
Birthweight, kg	11,211	3.4 (0.6)	3.6 (0.5)	<0.001
Gestational age, days	10,509	277.8 (15.1)	279.9 (12.3)	<0.001
Age at conscription, years	12,981	17.9 (0.8)	17.8 (0.5)	<0.001
Average mark at 16 years (1988–1997 graduates)	5,929			<0.001
Mark (1–5)		2.9 (0.8)	3.1 (0.7)	
*z* score		−0.270 (1.090)	0.002 (1.00)	
Overall mark at 16 years (1998–2009 graduates)	5,342			<0.001
Mark (0–320)		188.0 (61.6)	200.3 (56.6)	
*z* score		−0.185 (1.096)	0.001 (0.999)	
IQ at 18 years	10,735	95.2 (14.4)	97.6 (14.3)	<0.001

Continuous variables are expressed as mean (SD); categorical variables are expressed as *n* (%)

^a^ *n* with measure and included in analyses, 270,693

Table 2 shows the within-sibship, between-non-sibling and overall associations between maternal diabetes in pregnancy and offspring educational achievement at 16 years of age. Results were of similar magnitude for men who completed their compulsory education in 1988–1997 and those who graduated in 1998–2009. Overall, in the two cohorts as well as between non-siblings, maternal pregnancy diabetes was associated with lower educational achievement even when controlling for clustering within families and potential confounding by birth year, maternal age at birth, parity, education and maternal BMI in early pregnancy and mediation by gestational age and birthweight (models 1–4). However, within sibships we found no evidence of an association. For example, among non-siblings who graduated in 1998–2009, maternal pregnancy diabetes was associated with lower educational achievement in confounder-adjusted analyses (*β* = −0.09; 95% CI −0.14, −0.04, model 3), while the difference was in the other direction and the CI included the null value within sibships (*β* = 0.07; 95% CI −0.11, 0.25). We found strong statistical evidence across models that within-sibship associations differed from between-siblings associations (*p* ≤ 0.06 for all). Results of model 2 were virtually identical in the whole study population and in the subsample for which maternal early-pregnancy BMI data were available.

**Table 2:** Association of maternal diabetes in pregnancy with male offspring educational achievements at age 16 years, within sibships and between non-siblings

Outcome	Model	Number included in analyses	Mean difference (95% CI)			
			Within sibships	Between non-siblings	*p* value for difference^a^	Overall^b^
1988–1997 graduates						
Overall mean mark (birth year adjusted *z* score)	Model 1	391,545	0.04 (−0.13, 0.20)	−0.13 (−0.19, −0.08)	0.03	−0.12 (−0.17, −0.07)
	Model 2	391,545	0.05 (−0.12, 0.21)	−0.13 (−0.19, −0.08)	0.02	−0.12 (−0.17, −0.07)
	Model 4^c^	391,545	0.06 (−0.11, 0.22)	−0.15 (−0.20, −0.10)	0.009	−0.13 (−0.18, −0.08)
1998–2009 graduates						
Sum of top 16 marks (birth year adjusted *z* score)	Model 1	326,033	0.07 (−0.07, 0.20)	−0.10 (−0.15, −0.06)	0.009	−0.09 (−0.14, −0.05)
	Model 2	326,033	0.07 (−0.06, 0.21)	−0.11 (−0.15, −0.07)	0.005	−0.09 (−0.13, −0.05)
	Model 2^d^	268,829	0.07 (−0.11, 0.25)	−0.12 (−0.17, −0.07)	0.03	−0.11 (−0.15, −0.06)
	Model 3^d^	268,829	0.07 (−0.11, 0.25)	−0.09 (−0.14, −0.04)	0.06	−0.09 (−0.13, −0.04)
	Model 4^d^	268,829	0.07 (−0.11, 0.25)	−0.11 (−0.16, −0.06)	0.04	−0.10 (−0.15, −0.05)

All results are mean differences; the null value is 0

Model 1, adjusted for year of birth; model 2, adjusted for birth year, maternal age at birth, parity and education; model 3, adjusted for birth year, maternal age at birth, parity, education and BMI in early pregnancy; model 4, adjusted for birth year, maternal age at birth, parity, education, BMI in early pregnancy, gestational age and birthweight

^a^Obtained from the Hausman test, testing the null hypothesis that the within-sibling and between-non-sibling associations are identical

^b^The overall association in the population without control for fixed maternal characteristics, taking family clustering into account in the estimation of 95% CIs

^c^Not adjusted for early pregnancy BMI as it was not recorded

^d^ *n* reduced owing to missing data for early-pregnancy BMI

Table 3 shows the within-sibling, between non-sibling and overall associations of maternal diabetes in pregnancy and offspring IQ assessed at 18 years. Overall, with control for clustering within families, offspring IQ was on average 1.59 lower (95% CI −2.09, −1.08) in those whose mothers had diabetes in pregnancy compared with those who had not (model 1). Adjusting for potential confounders resulted in a slight attenuation (models 2 and 3), while inclusion of gestational age and birthweight slightly increased the effect size (model 4). Similar results were obtained from the between-non-sibling analyses. However, in within-sibling analyses, controlling for all shared characteristics, we found no strong evidence of an inverse association between maternal pregnancy diabetes and IQ in any of the models. In fact, effect estimates were all positive, though CIs included the null value. Results of model 2 for the whole population and for the subsample for which maternal early-pregnancy BMI was available were slightly different in size but not majorly so. There was moderate statistical evidence that within-sibling results differed from between-sibling results (*p* ≤ 0.09 for all).

**Table 3:** Association between maternal diabetes in pregnancy and male offspring IQ at mean age 18 years, within sibling groups and between unrelated individuals

Model	Number included in analyses	Mean difference in offspring IQ (95% CI) in men exposed to maternal pregnancy BMI compare to those unexposed			
		Within sibships	Between non-siblings	*p* value for difference^a^	Overall^b^
Model 1	664,871	0.92 (−0.49, 2.33)	−1.80 (−2.34, −1.26)	<0.001	−1.59 (−2.09, −1.08)
Model 2	664,871	0.94 (−0.47, 2.34)	−1.78 (−2.29, −1.27)	<0.001	−1.52 (−2.00, −1.04)
Model 2^c^	231,374	1.81 (−1.70, 5.31)	−1.41 (−2.17, −0.65)	0.07	−1.28 (−2.08, −0.53)
Model 3^c^	231,374	1.78 (−1.72, 5.29)	−1.15 (−1.91, −0.38)	0.09	−1.04 (−1.79, −0.30)
Model 4^c^	231,374	1.70 (−1.80, 5.21)	−1.36 (−2.12, −0.60)	0.08	−1.24 (−1.99, −0.50)

All results are mean differences; the null value is 0

Model 1, adjusted for birth year; model 2, adjusted for birth year, maternal age at birth, parity and education; model 3, adjusted for birth year, maternal age at birth, parity, education and BMI in early pregnancy; model 4, adjusted for birth year, maternal age at birth, parity, education and BMI in early pregnancy and gestational age and birthweight

^a^Obtained from the Hausman test, testing the null hypothesis that the within-sibling and between-non-sibling associations are identical

^b^The overall association in the population without control for fixed maternal characteristics, taking family clustering into account in the estimation of 95% CIs

^c^ *n* reduced owing to missing data for early-pregnancy BMI

## Discussion

In this large family-based study of men born in Sweden, we found that those exposed to diabetes in utero had a lower mean educational achievement upon completion of compulsory education at 16 years of age and mean IQ assessed at approximately 18 years of age at the conscription examination in standard multivariable analyses. The difference in IQ between men born to women with pregnancy diabetes and those born to women without pregnancy diabetes reported here (−1.52 IQ points) is somewhat smaller than that found in a UK-based cohort (ALSPAC) in which IQ was assessed at age 8 years (−4.85 for gestational diabetes and −2.24 for pre-existing diabetes) [8]. In that study we also found that maternal diabetes in pregnancy was associated with lower school achievements at 16 years in offspring.

While in the current study exposure to maternal diabetes in utero was associated with lower educational achievement and IQ in the overall cohort and between non-siblings, we found no evidence of this within sibships. In fact, the direction of association observed within sibships suggests that exposure to maternal diabetes in pregnancy is associated with higher educational achievement and IQ, though estimates were imprecise with wide CIs (which included the null value) due to the smaller sample size included in the fixed-effect model (restricted to siblings).

The fixed-effect model, which estimates the association within sibships, controls for characteristics that are fixed (i.e. similar or identical in siblings), including maternal genotype, family behaviour and early-life socioeconomic position. The different results from the overall and between-non-siblings analyses compared with within-sibling analyses suggested that similarities shared by siblings and controlled for in the sibling analysis, but not in the other models, are driving the modest associations observed in the cohort as a whole and between non-siblings. In other words, the findings of this study and others suggest that a direct effect of exposure to a diabetic milieu and/or perinatal complications resulting from maternal diabetes are unlikely explanations for the observed association between maternal diabetes in pregnancy and offspring cognitive outcome in the overall population.

An alternative mechanism (see Fig. 1) is that maternal genetic variants associated with diabetes are also associated with cognitive outcomes. These are then inherited by offspring and result in an association in non-siblings but not within sibships. However, we are unaware of any evidence supporting this proposed mechanism. On the contrary, in ALSPAC there was no association between maternal genetic risk score for greater fasting glucose concentrations and offspring IQ and a positive association between maternal genetic risk score for type 2 diabetes and offspring IQ [26]. This is in line with results here in the within-sibling analysis and those reported by Veena et al [10] and should be investigated further. The most likely explanation for our findings is that shared familial socioeconomic position accounts for the association between maternal pregnancy diabetes and offspring cognition seen overall and between non-siblings as IQ and education are socially patterned, as is diabetes [27, 28]. In our analysis we adjusted for maternal education, which is an indicator of socioeconomic position, but it is doubtful that it fully captures socioeconomic position and residual confounding is likely.

The main strengths of the present study are its large sample size and the use of a natural experimental approach in the form of a sibling study to explore associations of interest. This allowed us to determine whether associations are likely to be explained by familial confounding (maternal genetics or shared family behaviours) or by intrauterine effects. Potential limitations of sibling control studies have been postulated [29]; however, these have been based on simulations alone and extreme conditions that we think are unlikely here. A more likely limitation would be our inability to differentiate between type 1, type 2 and gestational diabetes because this information was not available from the Swedish Medical Birth Register. However, the majority of pregnancy diabetes is likely to be gestational diabetes—in the UK, 88% of pregnancies complicated by diabetes involve gestational diabetes [30]. In general, diabetes of any form would have similar effects in terms of exposing the fetus to higher levels of glucose; similar associations between pre-existing diabetes and gestational diabetes and offspring cardiometabolic outcomes have been reported [31, 32]. However, women with a known diagnosis before pregnancy may be better controlled than those who develop gestational diabetes. Indeed, in a previous study we found stronger inverse associations between gestational diabetes and offspring IQ at 8 years of age than between pre-existing diabetes and offspring IQ [8]. It is also possible that some unexposed siblings were in fact exposed to either undiagnosed diabetes or to relatively high (but not-diabetic) levels of maternal glucose, which would result in an underestimate of the association within sibships. Another limitation to this study is that only male offspring were included and, therefore, results may not necessarily generalise to female offspring.

In summary, our study suggests that the association between maternal pregnancy diabetes and lower offspring cognitive ability is likely to be driven by common shared familial characteristics and not by an intrauterine mechanism.

## Abbreviations

ALSPAC: Avon Longitudinal Study of Parents and Children
IQ: Intelligence quotient
